# Digital health tools and point solutions—pitfalls in population health program measurement

**DOI:** 10.3389/fdgth.2026.1785058

**Published:** 2026-06-09

**Authors:** Carolyn Langer, James Shaw, Wiljeana Glover

**Affiliations:** 1 Department of Family Medicine & Community Health, UMass Chan Medical School, Worcester, MA, United States; 2 Elevance Health, Woburn, MA, United States; 3 University of Toronto, Toronto, ON, Canada; 4 Babson College, Worcester, MA, United States

**Keywords:** point solution, population health, plan sponsor, user engagement, digital health procurement, digital health, eHealth

## Abstract

Digital health tools are generally poorly regulated and often lack strong research evidence, posing challenges for purchasers of point solutions such as employer groups and insurers. Point solutions, which are digital tools designed to manage health-related conditions, to promote wellness, and/or to drive member engagement, present unique challenges for purchasers. In this Review we provide a concise summary of 10 pitfalls in the interpretation of messaging from digital health vendors for purchasers seeking to make good investments in point solutions. We highlight existing population health research approaches that digital health vendors can employ to mitigate these pitfalls. We conclude with a recommendation that employers and health care entities gain clarity on goals related to outcomes they hope to achieve with digital health technologies, and make purchasing decisions based on a framework and objective data supporting the desired outcomes specifically related to those goals.

## Introduction

While digital health solutions have the potential to improve healthcare delivery and population health management, the rapid proliferation of digital health technology has been called the “Wild West” of healthcare (1). According to the IQVIA Institute for Human Data Science, about 337,000 consumer-facing digital health apps existed in 2024 (2). As healthcare costs continue to rise, employers, payers, and providers—particularly those providers transitioning to alternative payment and value-based care models, such as accountable care organizations (ACOs)—have embraced digital health and population health solutions to enhance access, mitigate medical expense trends, and improve health outcomes and quality (3–5). However, these decisionmakers often lack the framework or data to appropriately assess the range of digital health solutions (6, 7).

According to the U.S. Food and Drug Administration (FDA), “digital health technologies use computing platforms, connectivity, software, and sensors for health care and related uses. These technologies span a wide range of uses, from applications in general wellness to applications as a medical device…” (8). Many of these digital health vendors market to payers, employer groups, providers, and directly to consumers, offering products for a range of conditions and chronic diseases, such as musculoskeletal (MSK) disorders, infertility, women's health, weight management, diabetes, heart failure, oncology care, gastrointestinal disorders, behavioral health conditions, opioid use disorder (OUD), alcohol use disorder (AUD), and more (9). Point solutions are digital tools often developed by third party vendors and by payers typically to manage a targeted condition (e.g., asthma) or a set of related conditions (e.g., obesity, diabetes, hypertension, coronary heart disease) (10). Large employer groups regularly purchase multiple point solutions with which users interact for a range of aims, including wellness activities, condition-specific care, or chronic disease management, often through a digital app or personal computing device.

Despite the enthusiasm for digital health interventions, many digital health tools are poorly regulated, lack peer-reviewed published studies, and assert claims related to cost savings and quality that lack substantiation or supporting data (6). This paper highlights the major challenges in the assessment of digital health technologies and illustrates some common pitfalls in interpreting program outcomes and selecting digital health solutions. We then highlight existing population health research approaches that digital health vendors can employ to mitigate these pitfalls. Privacy and security—fundamental considerations in the implementation of digital health solutions—are not within the scope of this discussion. This article was written by two academics with expertise in policy and governance of digital health and by a physician with over 25 years of experience in managed care. Hypothetical examples in this article are based on actual occurrences in a range of managed care settings.

## Measuring outcomes and cost savings

Cost savings and return on investment (ROI) are important catalysts for the adoption of digital health solutions, although not always a purchaser's sole consideration. While traditional medical and pharmacologic interventions often undergo robust clinical studies prior to FDA clearance or approval and payer coverage determinations, few digital health solutions—particularly those categorized as general wellness products—undergo the same rigorous scrutiny. Section 520(o)(1)(B) of the Federal Food, Drug, and Cosmetic Act excludes FDA regulation of devices that incorporate software function when it is “intended for maintaining or encouraging a healthy lifestyle and is unrelated to the diagnosis, cure, mitigation, prevention, or treatment of a disease or condition.” (11) Additional FDA guidance defines low risk general wellness products excluded from FDA regulation as those products “intended for only general wellness use” and that “present a low risk to the safety of users or other persons.” (12) Further, this guidance explicitly states that a “product's inclusion under the general wellness policy…does not establish that it has been shown to be safe and/or effective for its intended use.” (12) Given that this burden often falls to purchasers, they should be aware of the following pitfalls in assessing health outcomes and cost savings.

### Pitfall 1: identifying the target population

Population health has been defined as “the health outcomes of a group of individuals, including the distribution of such outcomes within the group.” (13) The goals of a population health program should inform the selection of the target population and the appropriate digital health solution to offered to that population. Conventionally, goals may include one or more of the following: improving health outcomes; increasing access; closing gaps in care; promoting quality of care; reducing healthcare costs; improving health equity; enhancing operational efficiencies and employee productivity; bolstering employee recruitment, retention, and morale; and more. The target population should be meaningful, relevant, and impactable. For example, an employer implementing a musculoskeletal (MSK) digital health intervention to mitigate medical spend might optimize this goal by partnering with a point solution with a demonstrated ability to leverage predictive analytics and to meaningfully engage and impact high risk or emerging high risk individuals. In contrast, a solution that broadly markets directly to consumers with acute MSK injuries is likely to engage a higher percentage of low risk individuals with low severity MSK conditions, such as lumbosacral strain, which often resolve within a few weeks with limited intervention and are comparatively lower cost—resulting in lower cost savings and ROI. Furthermore, some conditions with high severity (e.g., infant with congenital heart defect, such as tetralogy of Fallot) may not be very impactable by a digital health solution targeting high cost claimants due to the unavoidable costs of expensive surgeries. The transparent inclusion of risk indices [e.g., (14)] and comorbidity indices [e.g., (15)] from population health and medical research within digital health vendor financial modeling (e.g., developing and illustrating financial models for a low-, medium-, and high-risk patient mix) may mitigate this pitfall, providing purchasers more clarity regarding potential health outcomes and cost savings.

### Pitfall 2: regression to the mean

**Figure 1:**
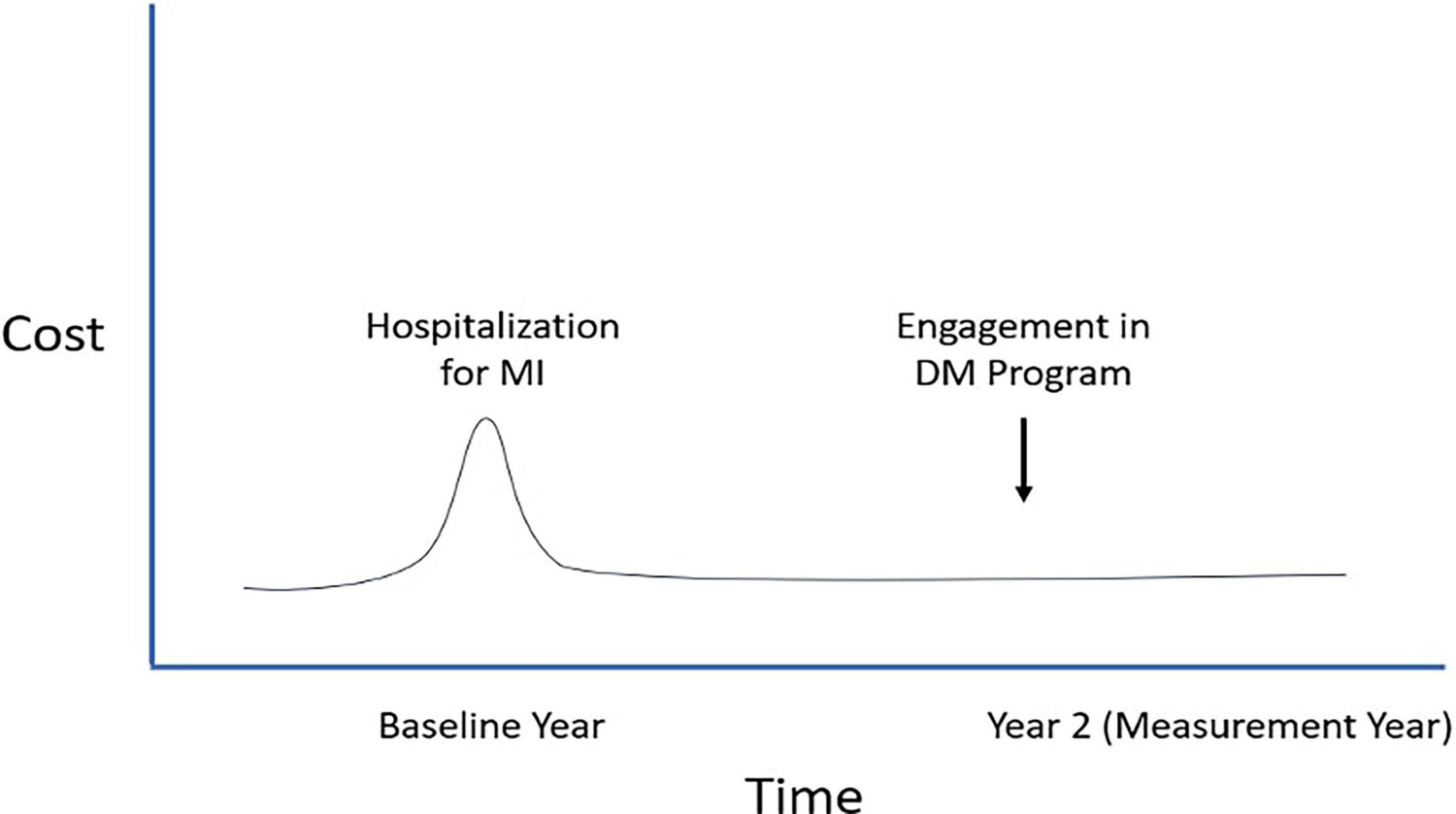
Regression to the mean.

Vendors may sometimes measure cost savings by comparing patients’ costs in the measurement year to their historical costs in the previous or baseline year. However, under the statistical principle of regression to the mean, one would expect later outcomes to be less severe and less costly compared to an earlier point in time, particularly for catastrophic or isolated resource-intensive events that are not likely to be recurring. This phenomenon is illustrated in Figure 1 in which the vendor identifies participants for a cardiovascular disease (CVD) management program based on a risk stratification process that targets patients who already had an event (e.g., myocardial infarction) in the prior year. The lower costs incurred by these patients in the measurement year after engagement in the CVD disease management (DM) program are not unexpected. These patients already experienced an acute event and hospitalization in the baseline year, and so health care costs would naturally fall in the following year, particularly because it is probable that the patients received a definitive intervention (e.g., cardiac stenting or CABG) during the acute event with ongoing pharmacologic treatment. Therefore, the vendor is likely to overstate the benefit of the digital health solution. Common strategies in population health studies to mitigate the impact of regression to the mean include the use of statistical analyses, such as analysis of covariance (ANCOVA), to adjust for initial baseline measurements when comparing pre- and post-intervention cost outcomes (16).

This figure was created by the authors for illustrative purposes.

### Pitfall 3: selection bias

Self-selection bias may occur if patients can select themselves into a treatment group, thereby weakening the generalizability of the association between the treatment and outcomes (17, 18). For example, some patients with acute MSK injuries who are more athletically active may have greater motivation to engage with an MSK digital health solution and demonstrate better outcomes than non-participants with MSK disorders. Similarly, a wellness digital health solution may attract participants who are more health-conscious and motivated to adhere to recommendations regarding diet, exercise, and well visits. In both instances, the participants in these two digital health programs will not necessarily be representative of the broader population eligible for these programs, and outcomes may potentially overstate the benefits. Common techniques to reduce selection bias include, but are not limited to, robust randomization, clear delineation of inclusion/excludion criteria, and sound analytical methodologies, such as weighted analysis, propensity score matching, or sensitivity analysis (19).

### Pitfall 4: confounder factors

Digital health and point solutions do not function in a vacuum. Typically, the purchaser of these services also has other vended programs and/or internal utilization management (UM) and care management programs. In some instances, these other vended and internal programs may also target and/or have an impact on the condition addressed by the digital health solution. For example, a digital health solution focused on gastrointestinal (GI) conditions may assert effectiveness after a claims analysis reflects lower GI-related costs. At the same time, the health plan may have instituted a new UM program that shifts GI procedures from more costly hospital outpatient settings to freestanding ambulatory surgery centers. Likewise, a digital health solution targeting heart failure patients may cite savings associated with reduced readmissions based on paid claims. However, the provider may have simultaneously implemented a new patient remote monitoring program for heart failure patients, or the payer may have implemented a new readmissions payment policy that bridges the claims from the readmission to those from the index (original) admission so that the readmissions will not appear as paid claims. Frequent methods to control for confounding bias include randomization, restriction (limits study subjects to individuals who are similar with respect to the confounder), matching, and statistical methods (stratification, multivariate analysis, propensity scores) (20). The absence of any strategies, such as multivariate analysis, to measure the impact of these confounding variables may distort the contribution of the digital health intervention and overstate savings.

### Pitfall 5: unmatched or poorly matched control group

To avoid selection bias to assess digital health solution efficacy, some vendors use a difference-in-differences technique when randomization of participants into intervention and control groups is not feasible. The difference-in-differences methodology longitudinally follows an intervention group and a control group, comparing differences in outcomes between the two groups [e.g., (21)]. This model assumes that intervention and control groups will follow parallel trends in outcome in the absence of the intervention before and after the intervention takes place. Purchasers should ensure that the control group is a fair comparator to the intervention group. For example, the use of a Medicaid population as the control group in a difference-in differences analysis of the impact of a high risk maternity program on a largely commercially-insured low-risk population may lead the vendor to overestimate the impact of the program on the intervention group if the Medicaid population experiences higher rates of maternal mortality and morbidity and higher NICU utilization.

### Pitfall 6: framing

**Figure 2:**
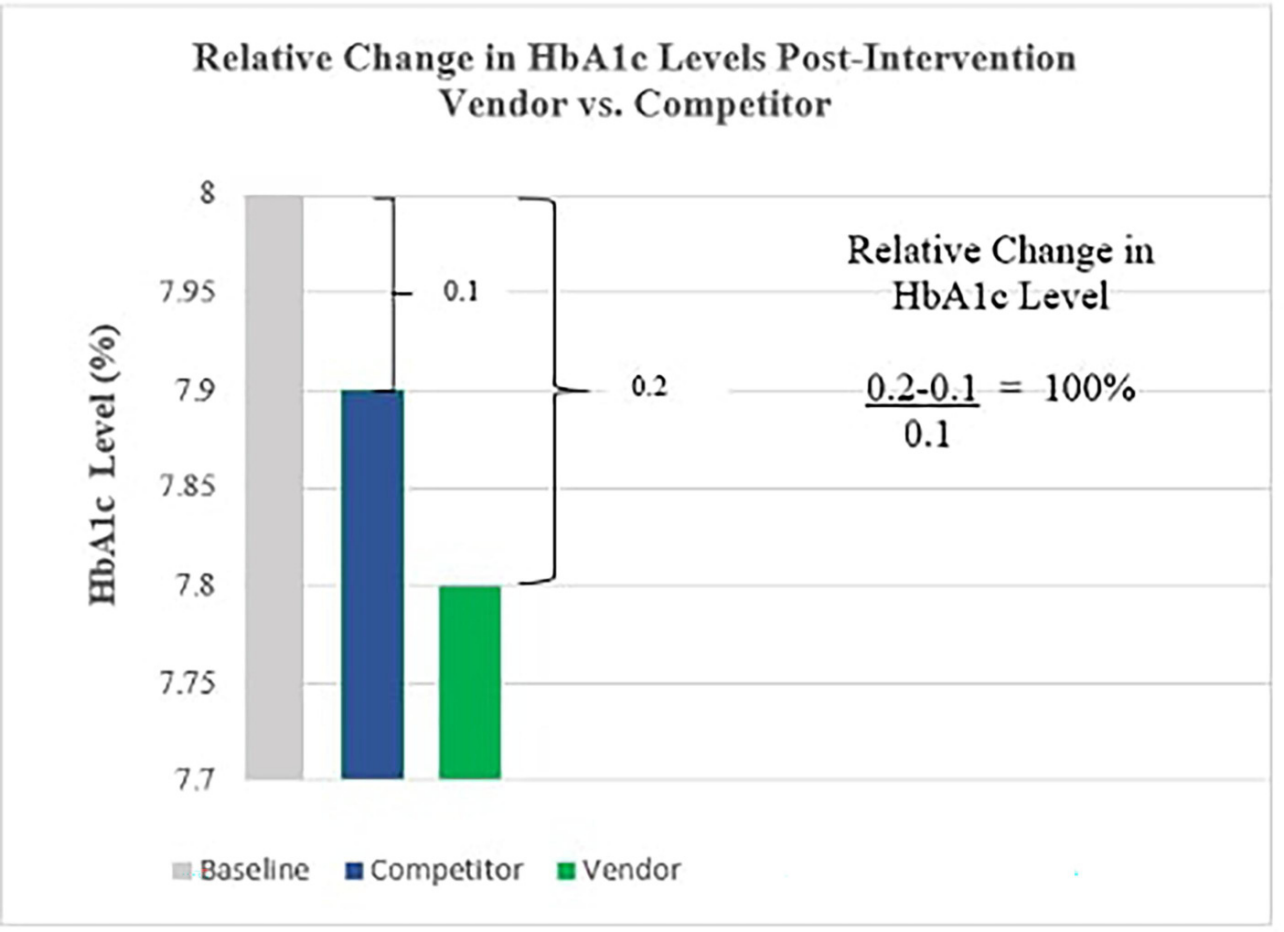
Relative change in hbAlc levels post-intervention vendor vs. competitor.

**Figure 3:**
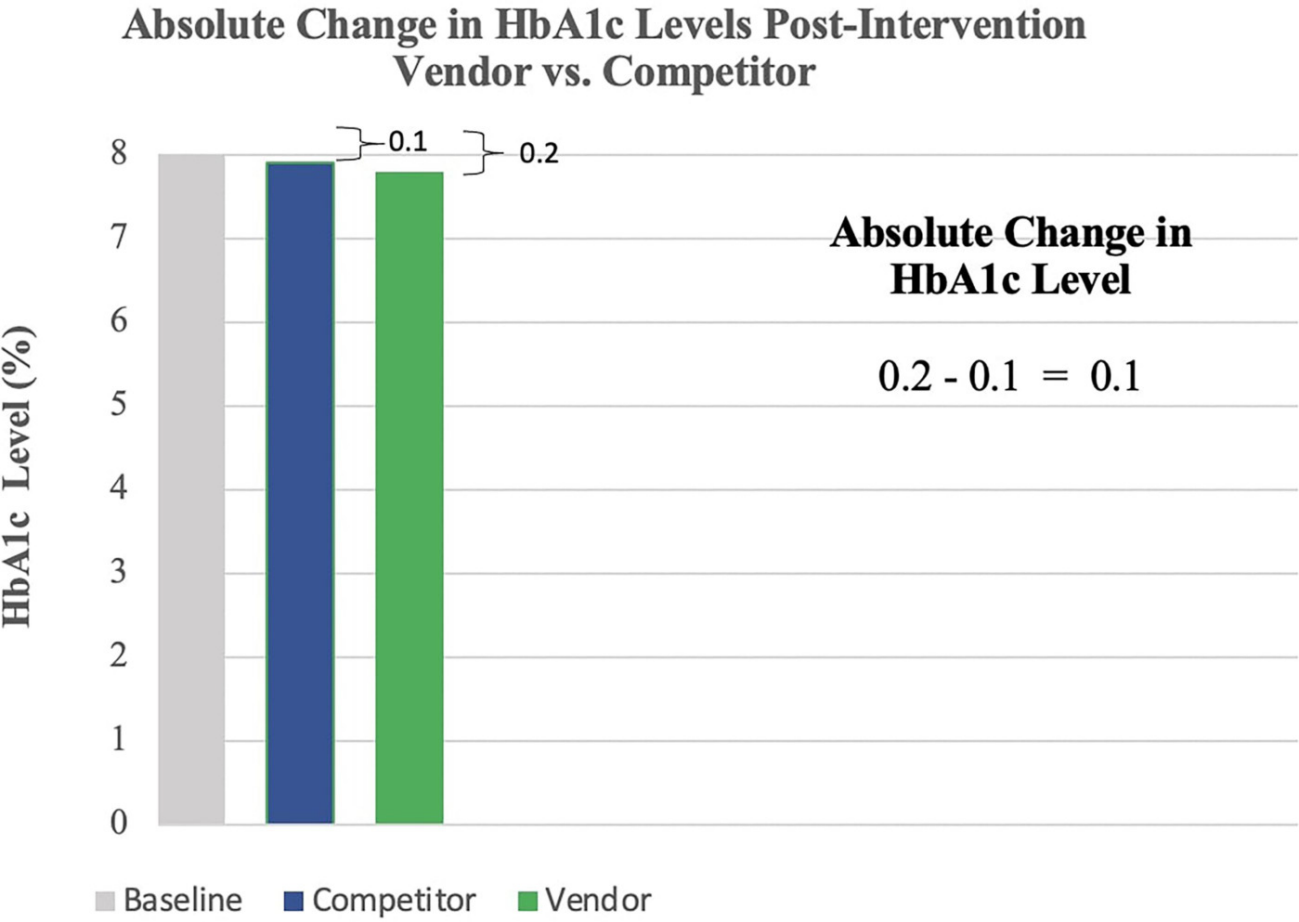
Absolute change in HbA1c levels post-intervention vendor vs. Competitor.

Framing describes the manner in which individuals make decisions based on how information is presented (22). Specifically, information presented as gains or losses lead to inconsistent decisions when considering the same problem because cognitive bias often leads individuals to opt for choices that are described more positively. For example, a vendor marketing an AI-driven digital app for screening malignant melanoma in the primary care setting may be more likely to emphasize that the tool accurately rules in or rules out melanoma in 40% of cases rather than highlight that findings are interderminate in 60% of cases. A vendor with a digital health solution for high risk diabetes patients may prefer to highlight health outcomes as a relative change rather than an as an absolute change. For instance, if the vendor's diabetes digital app has led to a drop in average hemoglobin A1c (HbA1c) levels from 8.0% to 7.8%, while a competitor's program has led to a drop in average HbA1c levels from 8.0% to 7.9%, the vendor's solution may be more appealing when the vendor frames the program's effectiveness as a 100% improvement in the change in HbA1c levels when compared to that of the competitor [(0.2%–0.1%)/0.1%] vs. framing an average absolute drop of 0.2% in HbA1c levels compared to the competitor's average drop in HbA1c levels of 0.1%. Visuals may also be misleading by amplifying the change even when not clinically significant (Figures 2, 3). To avoid framing bias, in addition to framing the data from a positive and negative perspective (22), request the underlying data (e.g., average absolute HbA1c decrease from 8.0% to 7.8% and from 8.0% to 7.9%, respectively), and visualize the information through diagrams, bar graphs, or charts.

### Pitfall 7: self-reported outcomes

Self-reported outcomes can be instrumental in understanding patients’ perspectives on their symptoms, side effects, functional status, and health-related quality of life (23, 24). To the extent available, vendors should use standardized tools, such as the SF-36, PHQ-2 or PHQ-9, GAD-7, etc., to capture patient-reported outcomes (25). While patient-reported outcomes and patient-reported experience are important measures to capture in assessing a digital health solution, self-reported information may not be appropriate in determining cost savings. For example, a telehealth or digital health solution that seeks to prevent avoidable emergency department (ED) visits measures cost savings by surveying patients following use of the program about the alternative care settings that the patients would have sought absent this intervention. If patients indicate that they would have visited the ED for their medical problem, the vendor categorizes these engagements as averted ED visits with the associated cost savings. Similarly, upon initially assessing patients, an MSK digital health solution asks patients if they are considering surgery. If patients respond “yes” and have not undergone surgery in a year, the vendor claims these cases as avoided surgeries with the associated cost savings. While self-reported outcomes may be appropriate to gauge pain levels, functional status, depression, and anxiety, the purchaser should question the validity of self-reported outcomes applied in cost savings estimates and should request peer-reviewed literature supporting this methodology [e.g., (26)]. Vendors and purchasers should also be aware that clinical efficacy related to the aforementioned pitfalls do not explain the working mechanisms of a digital health tool that ultimately contributes to purchaser outcomes, i.e., the adoption and use of digital health solutions. Issues related to adoption and use are highlighted in our final three pitfalls.

### Pitfall 8: defining patient engagement with point solutions and establishing patient engagement measures

**Figure 4:**
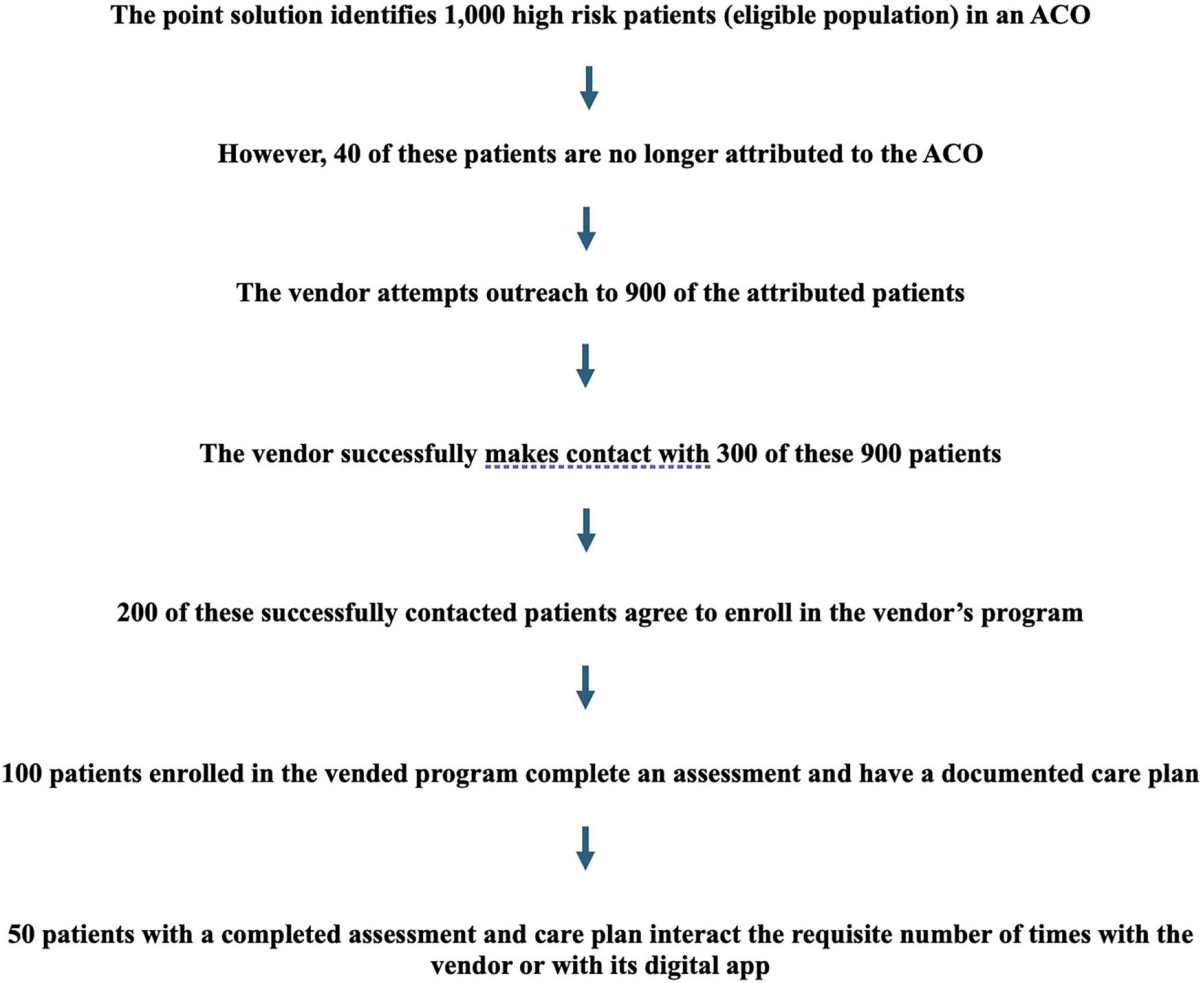
Examples of potential milestones in the user outreach and engagement process.

A key component of digital health solutions is patient engagement, activation, and/or self-management. Patient engagement is often included as a process measure in evaluating the effectiveness of digital health solutions. Yet, no standardized quantitative definition of patient engagement exists across the various digital health solutions. The digital health interventions literature notes that while engagement is a state of being involved with an object (in this case, the technology), the process of engagement includes behavioral (or physical), cognitive, and affect (or emotional) components that are influenced by context (27). That being said, the dual meaning of the phrase “patient engagement” poses challenges; patient engagement can also refer to the practice of engaging patient representatives in the design and delivery of health care policies and programs. This is a distinct usage of the phrase than that found in the digital health interventions literature, and as such, in this paper we refer to “user engagement” in relation to peoples’ uses of and interactions with digital health solutions. Vendors should seek to clarify their context, meanings, and measurement of user engagement when presenting solutions to purchasers, following recent examples, e.g., the TWente Engagement with Ehealth Technologies Scale (TWEETS) (28). The following examples of potential process measures for a diabetes management program illustrate the importance of understanding the context for metrics used by digital health vendors to calculate user engagement rates (see Figure 4 and Table 1):

**Table 1:** Diabetes management program engagement rates.

Patient engagement	Eligible population	Attributed population	Attempted contact	Successful contact	Agreement to participate in diabetes management program	Completed assessment & documented care plan	Interaction(s) with vendor or vendor tools
# Patients	1,000	960	900	300	200	100	50
Engagement Rate Based on Attributed Population	N/A	N/A	900/960 = 94%	300/960 = 31%	200/960 = 21%	100/960 = 10%	50/960 = 5%

As seen in the above example, rates can vary widely depending on the definition of user engagement. The decisionmaker should understand the proposed definition of user engagement employed by a digital health vendor or point solution and ensure that this definition aligns with the characteristics of the target population and the goals of the population health program. See Tables 2, 3 for key questions to address with digital health vendors:

**Table 2:** Potential challenges with outreach via telephone, text, chat, and/or email.

Potential barriers to patient outreach associated with these modalities
Some employer groups may limit or prohibit telephonic outreach Patients may elect a “do not call” designation if they are unwilling to receive telephone calls Phoning, texting, and emailing rely on having complete and accurate patient contact information from the employer, payer, or individual consumer Texting and emailing may require patient consent when these modes of communication are not conducted through a HIPAA compliant portal Chatting places the burden on the patient to proactively download an app onto a cell phone—extra steps that may further reduce the likelihood of connecting with the patient Individuals may not have access to the technology, may lack linguistic competence or health literacy, or may have certain disabilities that pose barriers to digitial accessibility

The decisionmaker may want to consider the available modes of outreach, any challenges the target population may have accessing the technology, and the likelihood of engagement with specific modalities when establishing the definition of user engagement for a particular program.

**Table 3:** Definitions commonly used by digital health vendors for “user engagement”.

Process measure	Definition
Attempted Outreach	The vendor attempts to connect with the patient through an agreed upon mode(s) of communication: a. The decisionmaker may opt to use this definition to ensure that the vendor is identifying and reaching out to the full target population; e.g., all high cost claimants over a specified dollar amount, all patients without an annual wellness visit, or all patients overdue for preventive screening tests. b. The decisionmaker and vendor should agree on the number of attempted outreaches and the times of day, days of the week and time span over which the vendor will attempt such outreach in order for the vendor's efforts to count as an “attempted outreach”
Successfully Outreached	The vendor is able to connect with the patient
Agreement to Enroll in Program	The patient agrees either verbally or in writing to enroll in the vendor's program (e.g., case management, health coaching, self-management programs)
Completed Assessment and Care Plan	completed assessment leads to a documented, individualized care plan: The patient asynchronously completes an online assessment, or the vendor synchronously administers an assessment to the patient most commonly via telephone or video call. The care plan documents meaningful goals to enable the patient to achieve improved health outcomes.
Interaction(s) with vendor or vendor tools	Proposed modes of interaction might include the following: a. The patient leverages the digital tool (e.g., downloads educational materials, logs a weight, tracks symptoms, walks through a self-guided meditation module or PT exercises, etc.). b. The patient interacts synchronously or asynchronously through an accepted mode of communication (video call, telephone call, email, text, chat) with a vendor representative.

The decisionmaker and vendor should agree on the number, frequency, and types of interactions that best align with the clinical condition, model of care, and goals of the program .

### Pitfall 9: integration with other population health management programs

Purchasers of digital health solutions frequently offer multiple digital health or point solutions to patients. Point solution fatigue can emerge when the purchaser's administrative burden of vetting, managing, and monitoring multiple solutions becomes unwieldy. Moreover, solutions that focus on single conditions run the risk of fragmenting patient care. At times, digital health solutions may overlap in scope, causing patient confusion. Point solutions often exist in siloes and fail to integrate with payers’ care management platforms or claims systems or with providers’ electronic medical records. They may not interface with the health plan's or provider's care management team or collaborate on co-managing cases. Because the scope of general wellness products excludes diagnosis and treatment, many point solutions will only interact with the patient and will not outreach to providers, placing the burden on patients to initiate discussions with their providers about their care. With greater awareness that a digital health tool is likely being implemented alongside other tools, vendors and purchasers alike may benefit from accounting for the multiple stakeholders engaged and the broader context in which the technology may be used (29).

### Pitfall 10: patient experience and health equity

Digital health solutions hold tremendous potential to improve health access and equity (30). For example, these tools can make care more available to individuals who live in rural areas, who have transportation or childcare barriers, who are homebound or have mobility issues, who require consultation with a specialist in a distant location, or who have mental health issues, such as anxiety or autism, that might impede the ability to travel or to sit in a medical office waiting room (31, 32).

Notwithstanding, purchasers and vendors should assess for any of the potential barriers described above and in the literature—access to technology (the digital divide), linguistic competency, and health literacy—to identify potential solutions (e.g., translation software) (33). Moreover, the purchaser should ensure that the tool is user friendly (the app is easy to install, the digital tool is simple to navigate, etc.), the vendor has reasonable customer service hours to assist patients with troubleshooting IT issues, and the content and delivery are effective in addressing the patient's issues. For example, a digital solution that raises palliative and end of life care as a potential service for a patient with newly diagnosed stage 1 breast cancer may be inappropriate and cause undue alarm. Finally, digital solutions that rely on algorithms to identify candidates for population health management programs should be assessed for bias. For instance, algorithms that ingest claims data to develop risk scores may be biased if certain underserved populations with historically worse access to health care are underrepresented in the claims database.

## Conclusion

Organizations often have limited bandwidth, and implementation of new digital health solutions consumes time and resources, even when vended. Therefore, purchasers should clearly articulate and prioritize the desired goals of their digital health strategy—such as enhanced cost savings, quality, access, health equity, patient experience, and/or employee productivity. Prior to implementation, the purchaser of digital health solutions should identify the optimal methodology and outcome measures to evaluate the intervention's effectiveness in meeting desired program goals. While there is no “silver bullet” in healthcare, attention to the highlighted concepts and potential pitfalls associated with the assessment and deployment of digital health solutions will enable payers, employers and providers to implement clinically and cost effective digital health solutions that promote a more comprehensive and evidence-based approach to population health management.

## Data Availability

The original contributions presented in the study are included in the article/supplementary material, further inquiries can be directed to the corresponding author/s.
